# The immunolocalization of cluster of differentiation 31, phalloidin and alpha smooth muscle actin on vascular network of normal and ischemic rat brain

**DOI:** 10.1038/s41598-022-26831-6

**Published:** 2022-12-24

**Authors:** Jia Wang, Yating Guo, Dongsheng Xu, Jingjing Cui, Yuqing Wang, Yuxin Su, Yihan Liu, Yi Shen, Xianghong Jing, Wanzhu Bai

**Affiliations:** Institute of Acupuncture and Moxibustion, China Academy of Chinese Medical Sciences, Beijing, 100700 China

**Keywords:** Neuroscience, Blood-brain barrier

## Abstract

Cluster of differentiation 31 (CD31), phalloidin and alpha smooth muscle actin (α-SMA) have been widely applied to label the cerebral blood vessels in the past years. Although CD31 is mainly used as endothelial marker in determining the cerebral capillaries, it seems likely that its labeling efficiency is closely correlated with the antibodies from the polyclonal or monoclonal one, as well as the conditions of blood vessels. In order to test this phenomenon, we compared the labeling characteristics of goat polyclonal anti-CD31 (gP-CD31) and mouse monoclonal anti-CD31 (mM-CD31) with those of phalloidin and α-SMA on the rat brain in health and ischemia/reperfusion (I/R) with the middle cerebral artery occlusion. By multiple immunofluorescence staining, it was found that gP-CD31 labeling expressed extensively on the cerebral capillaries forming the vascular networks on the normal and ischemic regions, but mM-CD31 labeling mainly presented on the capillaries in the ischemic region. In contrast to the vascular labeling with gP-CD31, phalloidin and α-SMA were mainly expressed on the wall of cortical penetrating arteries, and less on that of capillaries. By three-dimensional reconstruction analysis, it was clearly shown that gP-CD31 labeling was mainly located on the lumen side of vascular wall and was surrounded by phalloidin labeling and α-SMA labeling. These results indicate that gP-CD31 is more sensitive than mM-CD31 for labeling the cerebral vasculature, and is highly compatible with phalloidin and α-SMA for evaluating the cerebral vascular networks under the physiological and pathological conditions.

## Introduction

Cerebral blood vessels form the complex vascular network contributing to supply the sufficient oxygen and nutrients to satisfy the miscellaneous activities of the brain^1^. Unlike the blood vessels in other organs, cerebral blood vessels radially penetrate into the cerebral cortex presenting a structural and functional unique^2–4^. Since cerebral vascular disorder is closely correlated to various diseases^5–7^, how to select a proper biomarker to demonstrate the cerebral blood vessels has become an important issue in experimental study.

Cluster of differentiation 31 (CD31), also known as platelet-endothelial cell adhesion molecule-1 (PECAM-1), is an immunoglobulin superfamily member expressed on the surface of platelets, leukocytes and endothelial cells^8–12^. As the endothelial marker, various kinds of CD31 antibodies have been widely used in immunostaining the existing and developing blood vessels^10,11,13–15^. However, in our previous studies^13–16^, we found that the morphological characteristic of CD31 labeling was different according to the antibodies from the polyclonal or monoclonal one, as well as the conditions of blood vessels^13–16^.

In light of this phenomenon, we designed this study to firstly compare the labeling characteristics of goat polyclonal anti-CD31 (gP-CD31) and mouse monoclonal anti-CD31 (mM-CD31) in the healthy rat brain and the ischemia/reperfusion (I/R) rat brain with the middle cerebral artery occlusion (MCAO)^17–20^. Taking the advantage of the immunofluorescence staining, the difference of vascular labeling with gP-CD31 and mM-CD31 was systematically assessed on the cerebral cortex, including the normal region, as well as ischemic region, ischemic penumbra, and matching contralateral region.

In order to highlight the labeling characteristic of gP-CD31, comparatively, the other two kinds of vascular biomarkers, phalloidin and alpha smooth muscle actin (α-SMA) were also used to label the cerebral blood vessels. As it is well known, phalloidin is a specific probe for filamentous actin preferably expressing in smooth muscular and endothelial cells on the thick vascular wall^16,21–23^, while α-SMA presents in high amounts in smooth muscle cells on the vascular wall^16,21^. With the aid of multiple immunofluorescence staining and three-dimensional reconstruction techniques, we further analyzed the spatial correlation of vascular labeling with gP-CD31, phalloidin, and α-SMA from the cortical penetrating arteries to the intracerebral capillaries to obtain the cerebral vascular network in a three-dimensional pattern.

By these approaches, we expected not only to determine the labeling characteristic of CD31, but also to more effectively use it together with other vascular biomarkers for investigating the cerebral vascular network in morphological detail, which could provide additional and critical insights into the spatial correlation of multiple cellular components along the cerebrovascular tree under the physiological and pathological conditions.

## Results

### Distributional characteristics of the vascular labeling with gP-CD31 in the healthy and I/R brain

**Figure 1:**
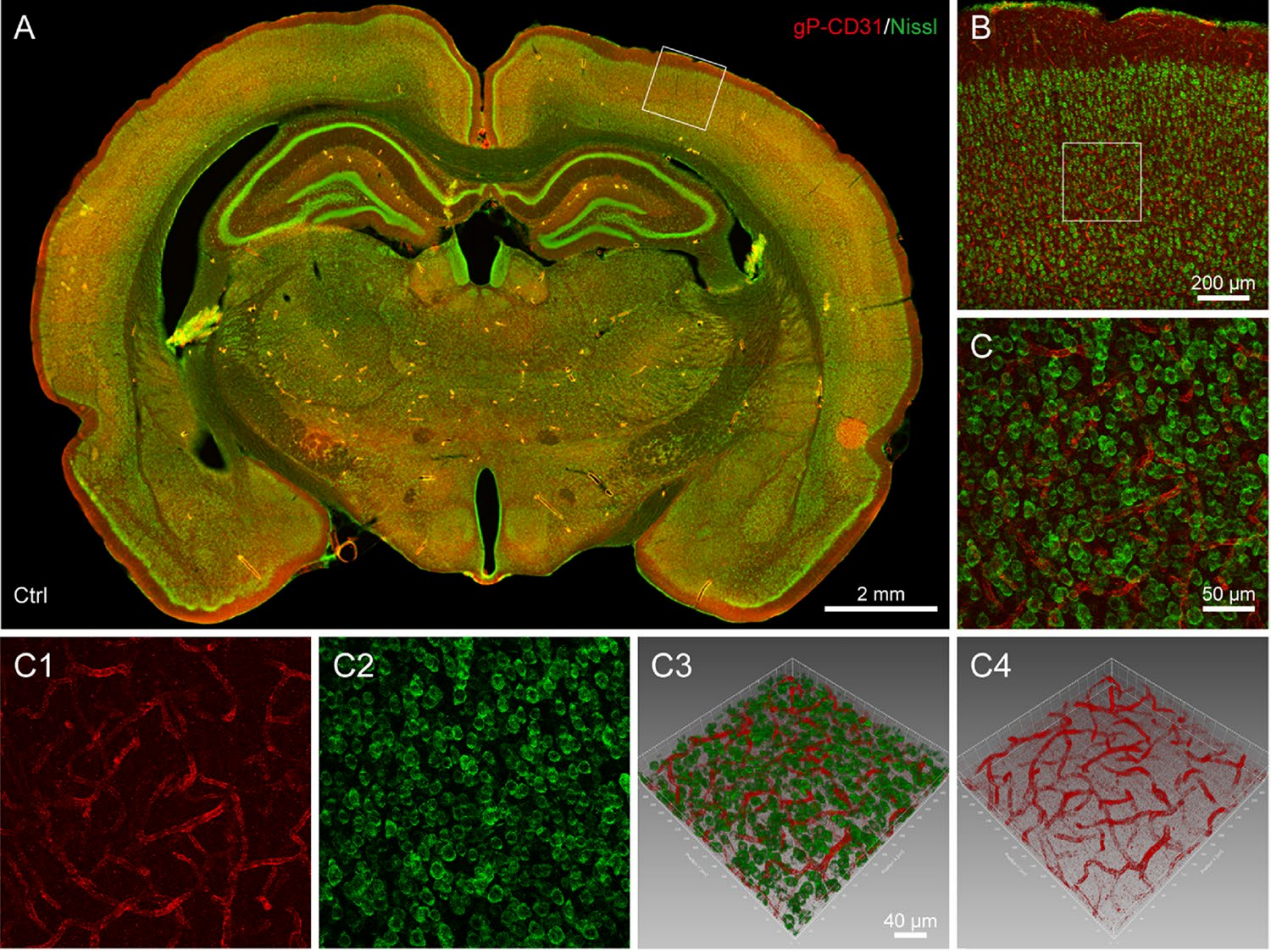
Distribution of vascular and cellular labeling with goat polyclonal anti-CD31 (gP-CD31) and Nissl in the healthy rat brain. (**A**) Representative montage view from the coronal section of healthy brain immunofluorescently labeled with gP-CD31 and Nissl. (**B**) Magnified photograph from panel A (box-indicated region) showing the distribution of vascular and cellular labeling in the cerebral cortex. (**C**–**C4**) The higher magnified photographs from the panel B (box-indicated region) showing the detailed vascular and cellular labeling (**C**) separately with gP-CD31 (**C1**) and Nissl (**C2**), and further adjusted in a sloping pattern with three-dimensional reconstruction (**C3**,**C4**). Scale bars: 2 mm in (**A**), 200 μm in (**B**), 50 μm in (**C**–**C2**), and 40 μm in (**C3**–**C4**).

**Figure 2:**
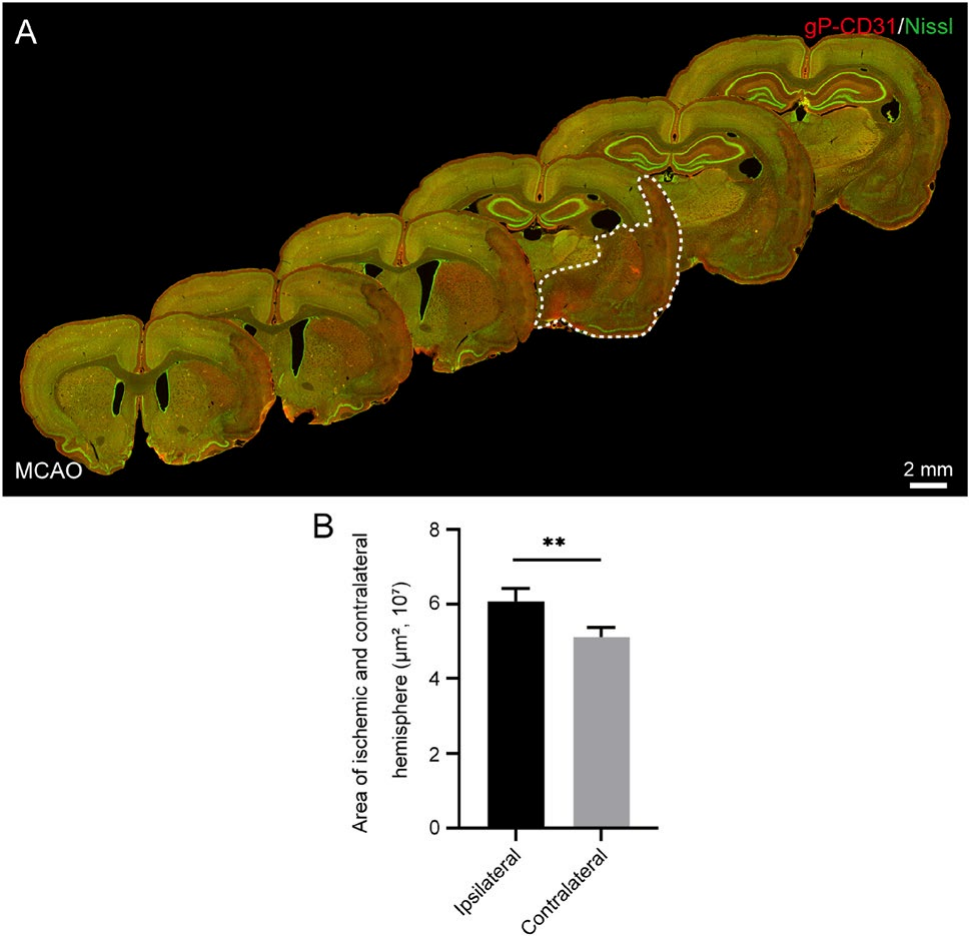
Regional alteration of the rat brain with ischemia/reperfusion. (**A**) Representative montage views from every 12th coronal sections of brain immunofluorescently stained with goat polyclonal anti-CD31 (gP-CD31) and Nissl showing the regional alteration induced by ischemia/reperfusion at vascular and cellular level. (**B**) Quantitative analysis of the areas of ischemic hemisphere and its contralateral hemisphere (n = 4 rats, **P < 0.05).

In order to show the regional characteristics of gP-CD31 labeled blood vessels in the healthy and I/R rat brains, the brain sections were counterstained with fluorescent Nissl staining for demonstrating the neuronal alternation on the cerebral tissue under the physiological and pathological conditions. On the background of Nissl staining, we focused our observation on the cortical region from 80-μm-thick slice, and traced the vascular labeling from the cortical penetrating arteries to the intracerebral capillaries to compare the vascular status on the normal, ischemic, penumbra, and matching contralateral regions (Figs. 1 and 2).

In the healthy rat brain, the cerebral structures, including cortex, thalamus, striatum, hippocampus and other regions, were symmetrically showed on the coronal section (Fig. 1A), in which gP-CD31 labeled blood vessels ran through the neurons forming cerebral vascular networks (Fig. 1B,C–C4).

**Figure 3:**
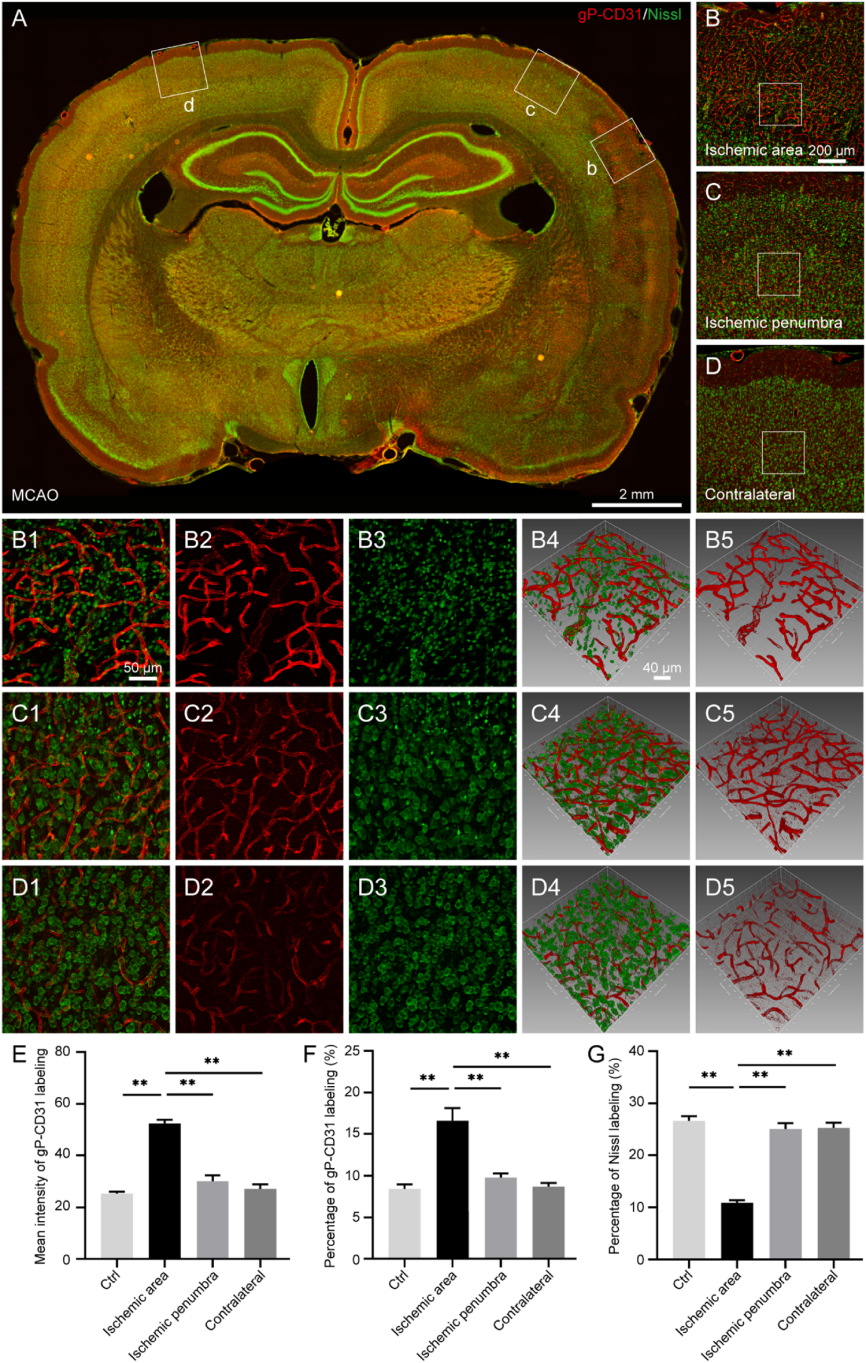
Distribution of vascular and cellular labeling with goat polyclonal anti-CD31 (gP-CD31) and Nissl in the rat brain with ischemia/reperfusion. (**A**) Representative montage view from the coronal section of ischemic brain immunofluorescently labeled with gP-CD31 and Nissl. (**B**–**D**) Magnified photographs from panel (**A**) (box-indicated regions) showing the distribution of vascular and cellular labeling in the cerebral cortex, including ischemic region (**B**), ischemic penumbra (**C**), and matching contralateral region (**D**). (**B1**–**B5**), (**C1**–**C5**), (**D1**–**D5**) The higher magnified photographs from the panel (**B**–**D**) (box-indicated regions) showing the detailed vascular and cellular labeling (**B1**,**C1**,**D1**) separately with gP-CD31 (**B2**,**C2**,**D2**) and Nissl (**B3**,**C3**,**D3**), and further adjusted in a sloping pattern with three-dimensional reconstruction (**B4**–**B5**, **C4**–**C5**, **D4**–**D5**). (**E**,**F**) Quantitative analysis of the labeling intensity (**E**) and the percentage (**F**) of gP-CD31 (n = 4 rats, 12 fields per region, **P < 0.05). (**G**) Quantitative analysis of the percentage of Nissl labeling (n = 4 rats, 12 fields per region, **P < 0.05). Scale bars: 2 mm in (**A**), 200 μm in (**B**–**D**), 50 μm in (**B1**–**D1**), (**B2**–**D2**), (**B3**–**D3**), and 40 μm in (**B4**–**D4**), (**B5**–**D5**).

In the I/R rat brain, the area of ischemic hemisphere was obviously larger than that of contralateral hemisphere, showing with the sign of edema (*P* < 0.05, Fig. 2A,B). In contrast to the contralateral and penumbra areas, the gP-CD31 labeling was more strongly expressed on the capillaries of ischemic area in parallel with neuronal loss (*P* < 0.05, Figs. 2, 3), which was more easily identified in the three-dimensional views (Fig. 3B4–D4,B5–D5).

### Labeling difference between gP-CD31 and mM-CD31 in the healthy and I/R rat brain

**Figure 4:**
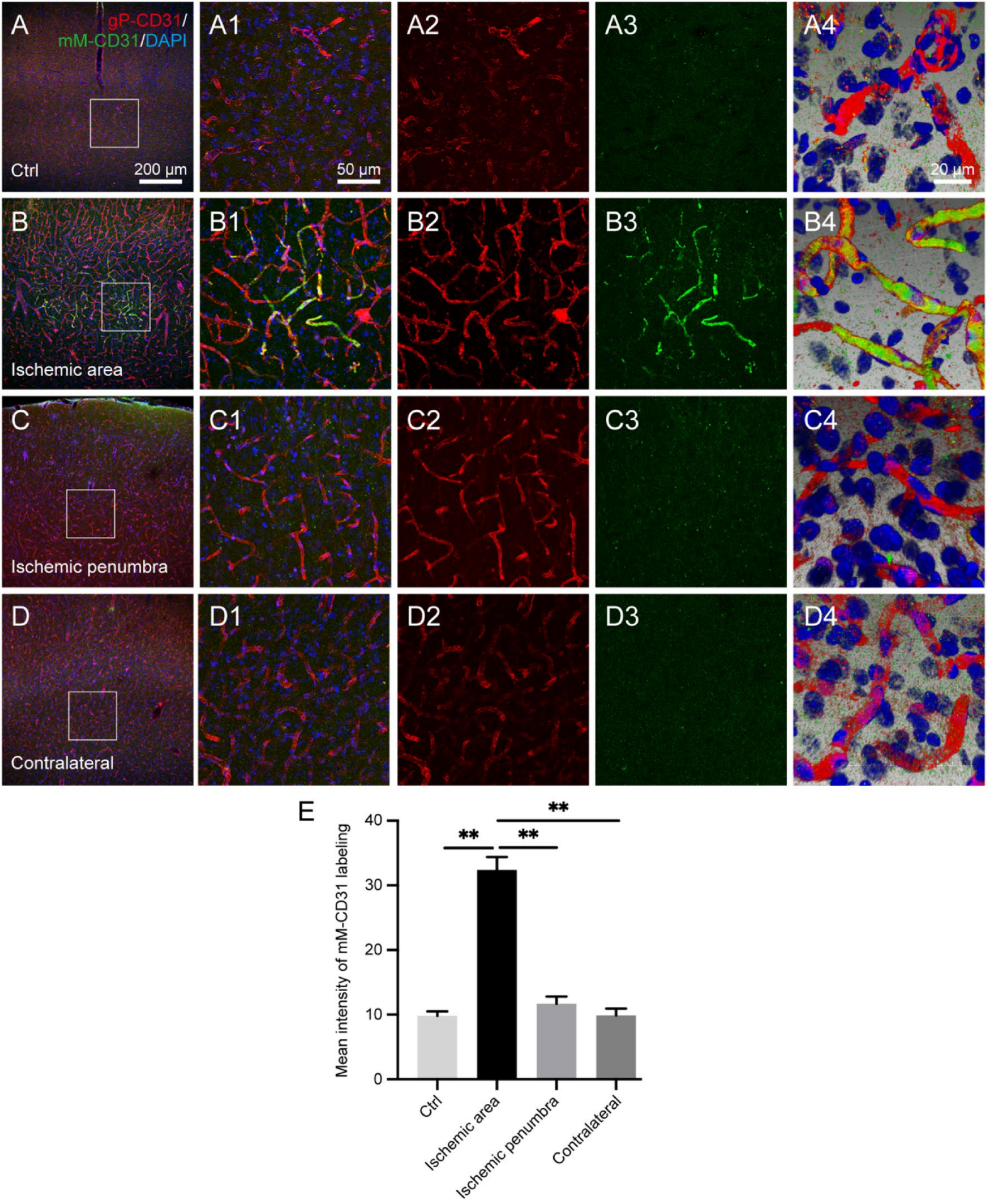
Comparing the distribution of cortical vascular labeling between goat polyclonal anti-CD31 (gP-CD31) and mouse monoclonal anti-CD31 (mM-CD31) in the healthy and ischemia/reperfusion rat brains. (**A**–**D**) Representative photographs from the cerebral cortex showing the difference of vascular labeling with gP-CD31 (red) and mM-CD31 (green) antibodies, including the normal region (**A**), ischemic region (**B**), ischemic penumbra (**C**), and matching contralateral region (**D**). (**A1**–**D1**), (**A2**–**D2**), (**A3**–**D3**) Corresponding magnified photographs from panels (**A**–**D**) (box-indicated regions) showing the detailed vascular labeling (**A1**–**D1**) separately with gP-CD31 (**A2**–**D2**) and mM-CD31 (**A3**–**D3**), and further adjusted with three-dimensional reconstruction showing the spatial relationship of vascular labeling with gP-CD31 and mM-CD31 in detail (**A4**–**D4**). The cellular nucleus was stained by DAPI (blue). (**E**) Quantitative analysis of the labeling intensity of mM-CD31 (n = 4 rats, 12 fields per region, **P < 0.05). Scale bars: 200 μm in (**A**–**D**), 50 μm in (**A1**–**D1**), (**A2**–**D2**), (**A3**–**D3**), and 20 μm in (**A4**–**D4**).

The labeling characteristics of gP-CD31 and mM-CD31 for cerebral vascular network were critically assessed in both healthy and I/R brains including the normal, ischemic, penumbra, and matching contralateral regions (Fig. 4). In contrast to vascular labeling with gP-CD31, mM-CD31 was preferentially expressed on the cerebral capillaries in the ischemic region where coexisted with that of gP-CD31 (Fig. 4B–B4), but there was less vascular labeling with mM-CD31 observed on the normal, penumbra, and matching contralateral regions (*P* < 0.05, Fig. 4A–A4,C–C4,D–D4,E). The spatial correlation of vascular labeling with gP-CD31 and mM-CD31 in ischemic region was clearly demonstrated in the three-dimensional views (Fig. 4A4,B4,C4,D4).

### Comparing the labeling characteristics of gP-CD31 and phalloidin

**Figure 5:**
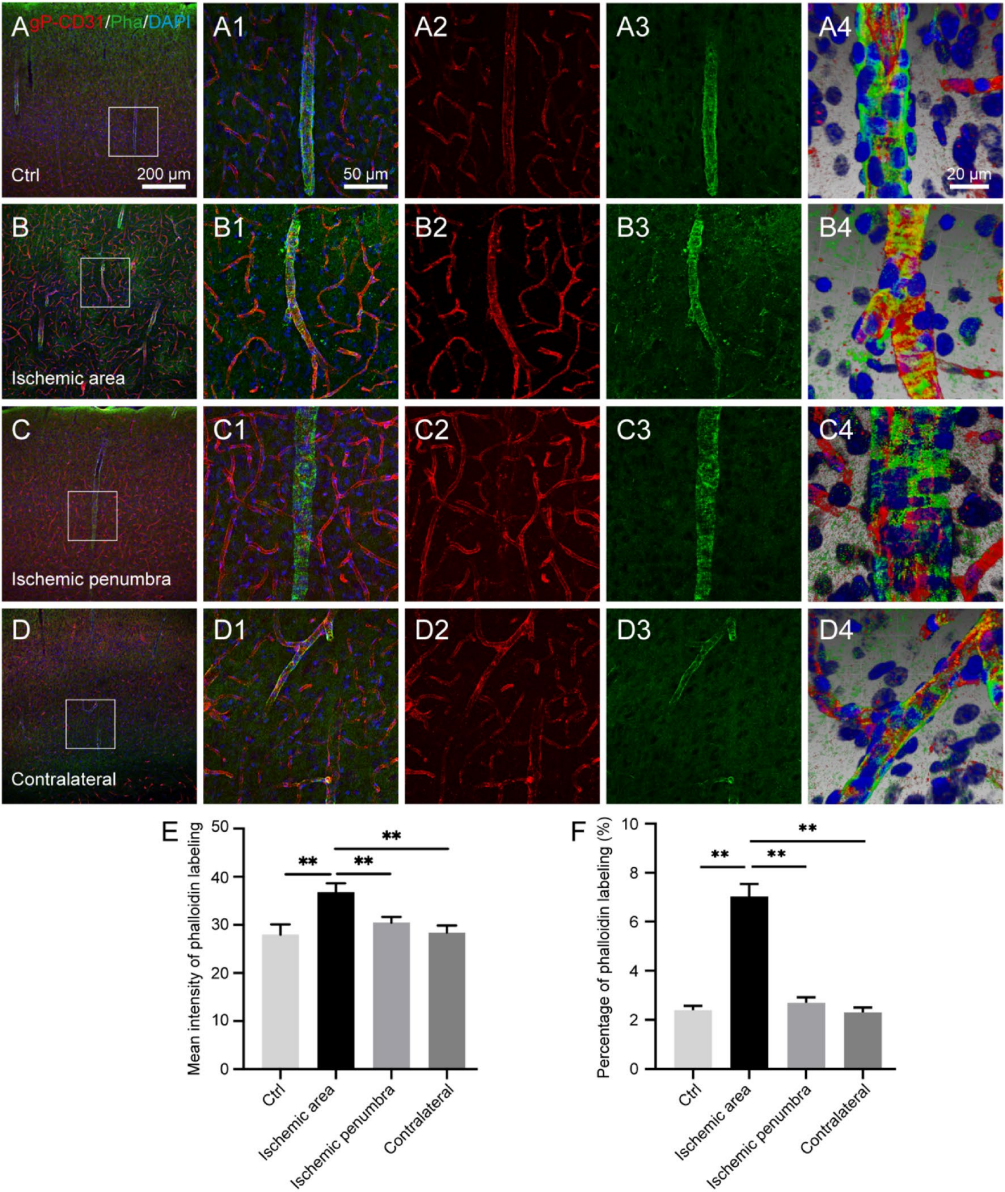
Comparing the distribution of cortical vascular labeling between goat polyclonal anti-CD31 (gP-CD31) and phalloidin (Pha) in the healthy and ischemia/reperfusion rat brains. (**A**–**D**) Representative photographs from the cerebral cortex showing the difference of vascular labeling with gP-CD31 (red) and phalloidin (green), including the normal region (**A**), ischemic region (**B**), ischemic penumbra (**C**), and matching contralateral region (**D**). (**A1**–**D1**), (**A2**–**D2**), (**A3**–**D3**) Corresponding magnified photographs from panels (**A**–**D**) (box-indicated regions) showing the detailed vascular labeling (**A1**–**D1**) separately with gP-CD31 (**A2**–**D2**) and phalloidin (**A3**–**D3**), and further adjusted with three-dimensional reconstruction showing the spatial relationship of vascular labeling with gP-CD31 and phalloidin in detail (**A4**–**D4**). The cellular nucleus was stained by DAPI (blue). (**E**,**F**) Quantitative analysis of the labeling intensity (**E**) and the percentage (**F**) of phalloidin (n = 4 rats, 12 fields per region, **P < 0.05). Scale bars: 200 μm in (**A**–**D**), 50 μm in (**A1**–**D1**), (**A2**–**D2**), (**A3**–**D3**), and 20 μm in (**A4**–**D4**).

For the vascular labeling, phalloidin was mainly expressed on the wall of cortical penetrating arteries, rarely on that of capillaries (Fig. 5). Comparatively, gP-CD31 labeling was located on the lumen side of vascular wall and wrapped with phalloidin labeling (Fig. 5A4–D4). Although the morphology of phalloidin labeled blood vessels was similar in both healthy and I/R brains, its expressing area and labeling intensity were larger and stronger in the ischemic region respectively (*P* < 0.05, Fig. 5E,F).

### Comparing the labeling characteristics of gP-CD31 and α-SMA

**Figure 6:**
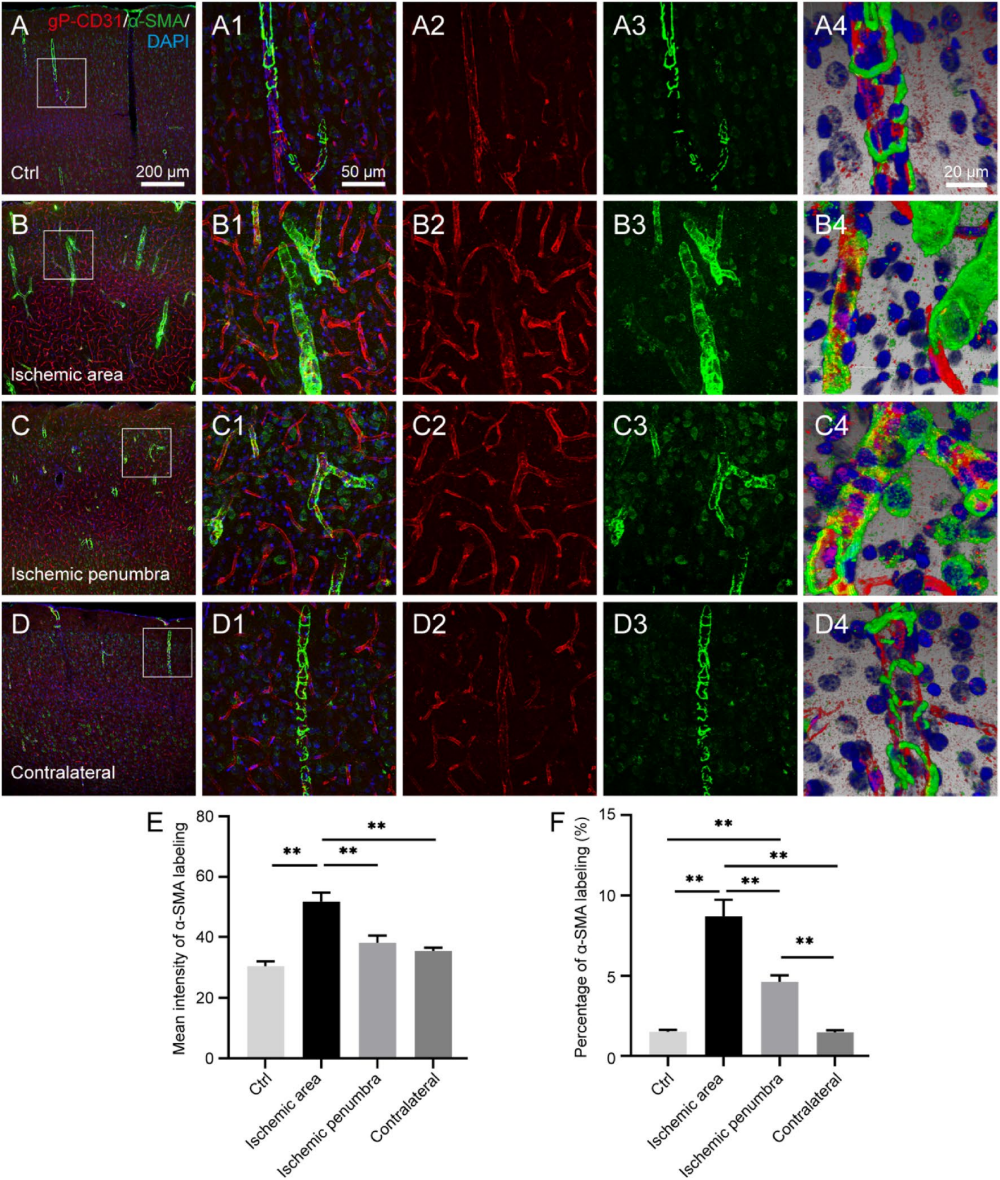
Comparing the distribution of cortical vascular labeling between goat polyclonal anti-CD31 (gP-CD31) and alpha smooth muscle actin (α-SMA) in the healthy and ischemia/reperfusion rat brains. (**A**–**D**) Representative photographs from the cerebral cortex showing the difference of vascular labeling with gP-CD31 (red) and α-SMA (green), including the normal region (**A**), ischemic region (**B**), ischemic penumbra (**C**), and matching contralateral region (**D**). (**A1**–**D1**), (**A2**–**D2**), (**A3**–**D3**) Corresponding magnified photographs from panels A-D (box-indicated regions) showing the detailed vascular labeling (**A1**–**D1**) separately with gP-CD31 (**A2**–**D2**) and α-SMA (**A3**–**D3**), and further adjusted with three-dimensional reconstruction showing the spatial relationship of vascular labeling with gP-CD31 and α-SMA in detail (**A4**–**D4**). The cellular nucleus was stained by DAPI (blue). (**E**,**F**) Quantitative analysis of the labeling intensity (**E**) and the percentage (**F**) of α-SMA (n = 4 rats, 12 fields per region, **P < 0.05). Scale bars: 200 μm in (**A**–**D**), 50 μm in (**A1**–**D1**), (**A2**–**D2**), (**A3**–**D3**), and 20 μm in (**A4**–**D4**).

In this study, α-SMA was also used for the vascular labeling. Similar to the phalloidin, α-SMA was also expressed on the wall of rat cortical penetrating arteries while less on capillaries (Fig. 6). The α-SMA labeling formed a disconnected layer surrounding the gP-CD31 labeling, which was more obvious under the three-dimensional views (Fig. 6A4–D4). Like gP-CD31, α-SMA was also expressed more and stronger in the ischemic area (*P* < 0.05, Fig. 6E,F).

### Spatial correlation of vascular labeling with gP-CD31, phalloidin, and α-SMA

**Figure 7:**
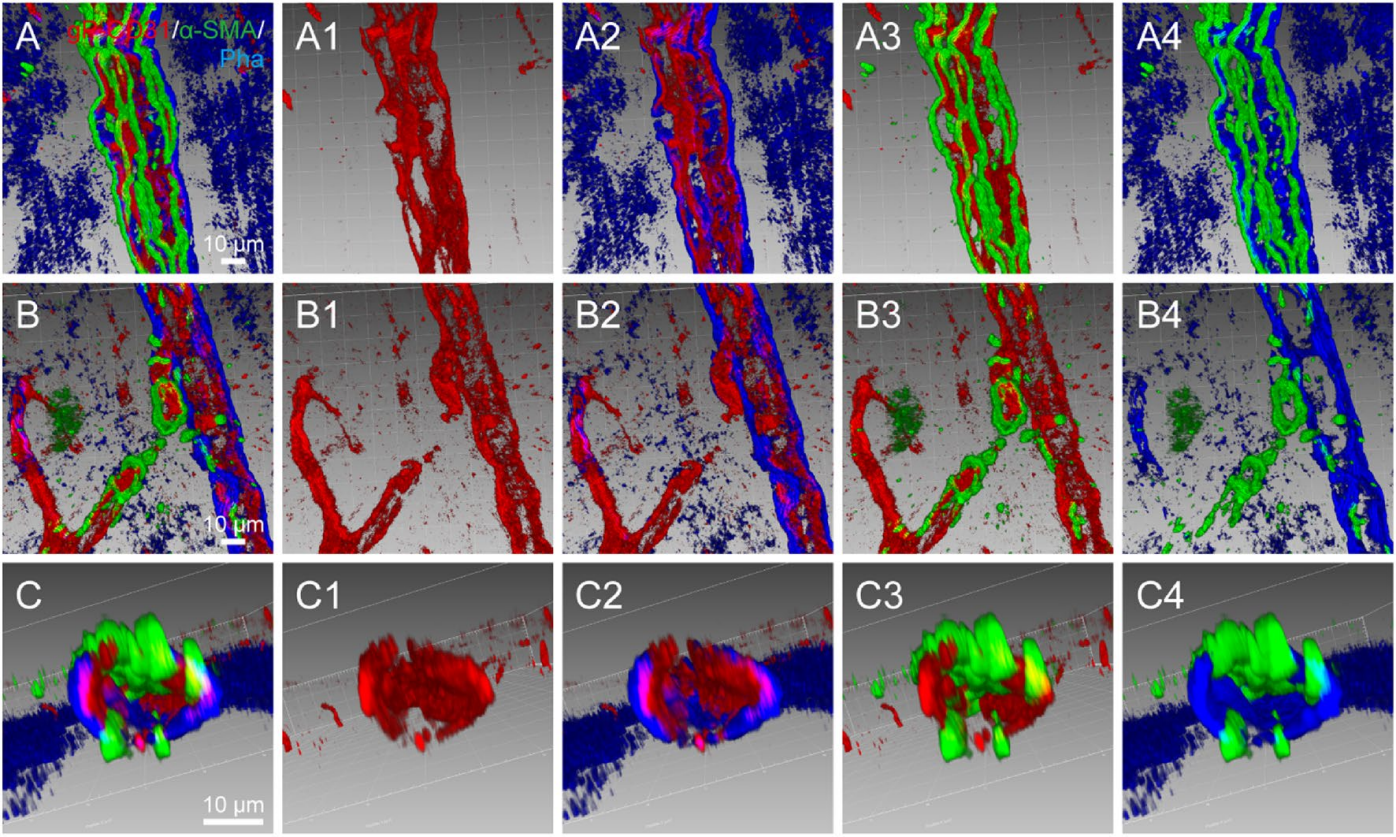
Spatial correlation of vascular labeling among goat polyclonal anti-CD31 (gP-CD31), phalloidin and alpha smooth muscle actin (α-SMA). (**A**–**C**) Representative adjusted images from the cerebral cortex showing the spatial correlation of vascular labeling of gP-CD31, phalloidin and α-SMA along the longitudinal axis of penetrating artery (**A**) and intracerebral capillaries (**B**), as well as with the transverse view of penetrating artery (**C**) in three-dimensional pattern. (**A1**–**A4**), (**B1**–**B4**), (**C1**–**C4**) Corresponding images from panels (**A**–**C**) showing the vascular labeling with gP-CD31 (**A1**–**C1**), gP-CD31 and phalloidin (**A2**–**C2**), gP-CD31 and α-SMA (**A3**–**C3**), phalloidin and α-SMA (**A4**–**C4**), respectively. Same scale bar for (**A**–**A4**), (**B**–**B4**), (**C**–**C4**), respectively.

Based on the above observations, the spatial correlations of vascular labeling among gP-CD31, phalloidin and α-SMA were further examined in the healthy rat brain and analyzed with three-dimensional reconstruction along the longitudinal axis of penetrating artery and intracerebral capillaries (Fig. 7A–A4,B–B4), as well as with the transverse view of penetrating artery (Fig. 7C–C4). It was further obvious that gP-CD31 labeling was surrounded by phalloidin and α-SMA labellings in the cortical penetrating arteries, but mostly independent in capillaries (Fig. 7A–A4,B–B4), while some of α-SMA labeling occasionally passed through the wall of the cortical penetrating arteries (Fig. 7C–C4). Although phalloidin and α-SMA were both expressed on the wall of cortical penetrating arteries, there was few colocalization to be observed under the three-dimensional views (Fig. 7C–C4).

## Discussion

In this study, we compared the cerebral vascular labeling properties of gP-CD31 with that of mM-CD31, as well as with those of phalloidin and α-SMA on the rat brains in health and I/R. Taking the advantage of multiple immunofluorescence staining on the thick brain sections, we provided histochemical evidence to show that gP-CD31 is superior to mM-CD31 on the vascular labeling in the normal and ischemic regions, and is highly compatible with phalloidin and α-SMA for insight into the cerebral vascular network in rat brain. These results indicate that the present technological approach should be beneficial in investigating the detailed morphology of cerebral vascular network under the physiological and pathological conditions, and has the potential to assess novel therapeutic interventions of cerebral vascular disorders.

### Technological consideration

With the development of microscopic techniques, multiple immunofluorescence staining has been the standard procedure for investigating morphological and molecular aspects in the sliced tissues for several decades^24–28^. In line with this field, we adopted the techniques of panoramic tissue slice scanner and laser scanning confocal microscope in this study. The former can catch the overall view of tissue section and the latter can take the clear image from the thick tissue^29,30^. Taking these advantages, we can simultaneously obtain the labeling information from two dimensions to three dimensions^29,30^. Based on this consideration, the regional characteristics of gP-CD31 labeling were firstly assessed together with fluorescent Nissl in the healthy and I/R rat brains. Beyond the 2,3,5-triphenyltetrazolium chloride (TTC) staining for evaluating the changes of cerebral infarction volume, Nissl staining can not only clearly show the ischemic area, but also demonstrate its morphological alteration at cellular level^13,14^. On the background of fluorescent Nissl staining, it was clear that gP-CD31 labeling formed the cerebral vascular network among the neurons in whole brain specimens under the physiological and pathological conditions. In contrast to the gP-CD31 labeling, the mM-CD31 labeling was mainly detected on the capillaries in the ischemic region. Therefore, it limits the application of mM-CD31 to combine with the other bio-markers for the vascular labeling. Comparatively, our present results indicate that gP-CD31 is a better candidate together with phalloidin and α-SMA for labeling the cerebral vascular networks in the three-dimensional views in rat.

### Monoclonal versus polyclonal antibodies of CD31

As it is known, antibodies are the key players in the immunohistochemistry to bind their corresponding target antigens. No matter what kinds of antibodies, monoclonal or polyclonal, their ability determined by the degree of affinity and specificity to bind an antigen^31–37^. In general, monoclonal antibody is regarded as an antibody of single and unique specificity for their intended targets, serving as a powerful tool for labeling macromolecules and cells^38,39^, and comparatively polyclonal antibody can detect a multiplicity of epitopes and recognize antigen from different orientations^31^, which may be important in certain assays where the detection of an analyte would be compromised by the use of a single epitope^39^. In addition, polyclonal antibodies are also more stable over a broad pH and salt concentration, and have better specificity than monoclonal antibodies^31^.

In the case of CD31, it has been widely used as an endothelial marker in determining microvascular density^40,41^. Although gP-CD31 labeling and mM-CD31 labeling were co-localized on the cerebral capillaries in the ischemic region (Fig. 4B, Supplementary Fig. 1), in contrast to the gP-CD31 labeling, the mM-CD31 labeling was less detected in the normal, ischemic penumbra, and matching contralateral regions. For the cerebral vasculature, gP-CD31 was expressed more extensively than that of mM-CD31, which was in consistent with the previous studies (see Supplementary Table 1). According to the specification of antibodies, gP-CD31 is a 130 kDa type I transmembrane glycoprotein adhesion molecule in the immunoglobulin superfamily^42,43^, its immunogen is mouse myeloma cell line NS0-derived recombinant mouse CD31/PECAM-1 (Glu18-Lys590, Accession # Q08481). And its expression is restricted to cells involved in circulation, platelets, monocytes, neutrophils, lymphocyte subsets, especially endothelial cells^42,43^. In contrast, mM-CD31 is an antibody approximately 130 kDa, which is expressed on platelets and leukocytes, and primarily concentrates at the borders between endothelial cells^44,45^. However, the underlying mechanism of the difference between both antibodies still remains elusive.

### Difference of vascular labeling between gP-CD31, phalloidin, and α-SMA

On the basis of gP-CD31 labeling, cerebral blood vessels were also successfully labeled with phalloidin and α-SMA. Along the cerebrovascular tree, three kinds of vascular labeling were located orderly on the vascular wall closely without coexisting between one another. This result supports the idea that the cerebrovascular tree is constituted by different kinds of vascular cells, and these cellular components were varied from the cortical penetrating arteries to the intracerebral capillaries^46^. Firstly, gP-CD31 labeling is located on the lumen side of the wall of cortical penetrating arteries and their main branches are surrounded with phalloidin and α-SMA labeling, which implies that gP-CD31 labeled endothelial cells stand on the first line to respond the harmful effects of cerebrovascular insufficiency^46^. Moreover, gP-CD31 labeling takes predominant advantage in the intracerebral capillaries, where phalloidin and α-SMA labeling is less detected, which suggests that gP-CD31 labeled endothelial cells are distributed more extensively than the vascular cells labeled with phalloidin and α-SMA. Therefore, gP-CD31 is not only suitable for labeling the cerebral vascular network, but also highly compatible with phalloidin and α-SMA for demonstrating the sub-types of vascular cells from the cortical penetrating arteries to the intracerebral capillaries in the health and disease.

### Potential application

According to the quantitative analysis of vascular labeling, gP-CD31 was expressed more strongly on the capillaries in the ischemic region associated with the neuronal degeneration. Under the physiological condition, endothelial, neuronal, and glial cells are interconnected for contributing to brain homeostasis^46–49^. However, our understanding of multicellular alteration associated with the I/R brain is still incomplete. Under the pathological condition, the precise role of higher expressed gP-CD31 in the ischemic region, whether detrimental or beneficial, remain poorly understood. It might be associated with the promotion of endothelial cell proliferation, migration, capillaries formation, vascular permeability or angiogenesis^48^. Higher permeability can lead to the edema of I/R hemisphere with neuronal and glial alteration, directly causing brain parenchyma lesions^13,50^, which is supported by our present histological evidence. Conversely, angiogenesis is a crucial process for neural repairing during I/R or other vascular injury in the brain, although the cerebral vasculature is quiescent in the adult animals under the normal condition^51^. Previous studies have shown that endothelial cells are mitotic and can generate new vessels in response to brain injury, and its proliferation occurs from 12 h after stroke and continues for a number of weeks^3,52^. However, our present observation is limited on the spatial alteration of vascular labeling at one time-point in the I/R brain, and the temporal changes of proliferation with endothelial cells remain to be determined in the same experimental model. Whether enhanced gP-CD31 expression could be the histochemical evidence for evaluating the angiogenesis, it might be an important issue for the further exploration in the future study. In addition, our results suggest that the combination of the multiple immunofluorescence staining with three-dimensional reconstruction techniques on the thick tissue sample is a beneficial approach for demonstrating the vascular alteration within I/R brain in a three-dimensional view, and it is reasonably used to show the vascular disorder with other diseases in histochemical detail^5–7^.

In summary, we have compared the vascular labeling characteristics of gP-CD31, mM-CD31, phalloidin and α-SMA on the cerebral blood vessels in this study. The results indicate that gP-CD31 is more sensitive than mM-CD31 for vascular labeling in both healthy and injured rat brain. Furthermore, our results suggest that gP-CD31 can be an ideal candidate, separately or together with other vascular, neural or glial bio-markers, for revealing the vascular network or neurovascular unit in the rat brain under the physiological and pathological conditions.

## Materials and methods

### Animals

Eight healthy male Sprague–Dawley rats (weighting 200 ± 20 g) were obtained from the National Institutes for Food and Drug Control (license number SCXK (JING) 2017-0005). Animals were housed in temperature- and humidity-controlled rooms (25 ± 1 °C; 45 ± 5%). All experimental procedures were approved by the ethics committee at China Academy of Chinese Medical Sciences (No. D2018-09-29-1), and performed in accordance with the National Institutes of Health Guide for the Care and Use of Laboratory Animals (National Academy Press, Washington, D.C., 1996).

### Animal model of ischemia/reperfusion with MCAO

Rats were randomly assigned to control and model groups (n = 4). The rats in I/R model group were conducted with MCAO. Four rats were anaesthetized with tribromoethanol solution (150 mg/kg) via intraperitoneal injection. Briefly, the right common carotid artery and external carotid artery were exposed, after ligating the external carotid artery, the internal carotid artery was carefully separated and a small incision was then cut on the right common carotid artery. After that, a 0.28 mm nylon filament was introduced into the distal right internal carotid artery for blocking the right middle cerebral artery until a slight resistance was felt at approximately 18 mm from the carotid artery bifurcation which ensured the occlusion at the origin of the middle cerebral artery. The filament was fastened around the distal of the right common carotid artery. After 1.5 h of occlusion, the filament was gently pulled out about 10 mm to allow complete blood reperfusion of the ischemic area^15,20^. No operations were performed on the rats in control group.

### Perfusion

After 24 h survival, the rats in control and I/R groups were anesthetized with an overdose of tribromoethanol solution (250 mg/kg) via intraperitoneal injection, and then transcardially perfused with 0.9% sodium chloride solution (100 mL) followed by 4% paraformaldehyde (PFA) in 0.1 mol/L (M) phosphate buffer (PB, pH 7.4, 300 mL). After perfusion, the brain was carefully dissected out and post-fixed for 2 h, then cryopreserved in 0.1 M PB containing 25% sucrose at 4 °C overnight.

### Section

Serial coronal sections of brain were cut at the thickness of 80 μm on a sliding microtome system (Yamato, REM-710) and collected in order in a six-hole Petridish with 0.1 M PB (pH 7.4).

### Multiple immunofluorescence staining

The five kinds of immunofluorescence staining were performed in this study, including ① gP-CD31 + Nissl; ② gP-CD31 + mM-CD31 + 4′,6-diamidino-2- phenylindole (DAPI); ③ gP-CD31 + phalloidin + DAPI; ④ gP-CD31 + mouse anti-α-SMA + DAPI; ⑤ gP-CD31 + phalloidin + mouse anti-α-SMA. The sections were incubated in blocking solution containing 3% normal donkey serum and 0.5% Triton X-100 in 0.1 M PB (pH 7.4) for 0.5 h at room temperature. After that, the sections in each group were orderly incubated with primary antibodies of gP-CD31 (1:1000, AF3628, R&D), mM-CD31 (1:500, ab24590, Abcam), mouse anti-α-SMA (1:1000, a2547, Sigma) corresponding to each group at 4 °C for overnight. On the next day, the sections were washed in 0.1 M PB (pH 7.4) and incubated with corresponding secondary antibodies with donkey anti-goat Alexa Fluor (AF) 594 (1:500, Thermo Fisher, USA) and donkey anti-mouse AF488 (1:500, Thermo Fisher, USA). During this procedure, Nissl 500/525 (1:1000, Thermo Fisher, USA) was used in group ①, phalloidin 488 (1:500, Thermo Fisher, USA), phalloidin 647 (1:500, Thermo Fisher, USA) and DAPI (1:50,000, Thermo Fisher, USA) were used in groups of ③, ⑤ and ②–④ respectively for counter staining for two hours. After that, the samples were washed with 0.1 M PB thoroughly and coverslipped with 50% glycerin. The primary and secondary antibodies, and other biomarkers used were listed in Table 1.

**Table 1:** List of primary and secondary antibodies, and other bio-markers.

Antibodies and other bio-markers	Host	Dilution	Producer	Cat. number
**Primary antibodies**				
CD31	Goat polyclonal	1:1000	R&D	AF3628
CD31	Mouse monoclonal	1:500	Abcam	ab24590
α-SMA	Mouse monoclonal	1:1000	Sigma	a2547
**Secondary antibodies**				
Donkey anti-goat AF594	–	1:500	Thermo Fisher	A11058
Donkey anti-mouse AF488	–	1:500	Thermo Fisher	A21202
**Others**				
Nissl 500/525	–	1:1000	Thermo Fisher	N21480
Phalloidin 488	–	1:500	Thermo Fisher	A12379
Phalloidin 647	–	1:500	Thermo Fisher	A22287
DAPI	–	1:50,000	Thermo Fisher	D3571

*α-SMA* alpha smooth muscle actin, *DAPI* 4′,6-diamidino-2-phenylindole.

### Observation

The samples were firstly scanned with the panoramic tissue slice scanner (VS120, Olympus, Japan). Accordingly, the selected cortical regions were further observed and recorded by using a laser confocal imaging system (FV1200, Olympus, Japan). Single image (10 ×, 1260 μm × 1260 μm) was scanned at a resolution of 1024 × 1024 pixels (pinhole = 152 μm), and a series of images (about 40 images were obtained with Z-stacks in the interval of 2 μm/step) in magnificent view (40 ×, 310 μm × 310 μm) were captured at a resolution of 640 × 640 pixels (pinhole = 105 μm). Three-dimensional reconstruction of the cerebral blood vessels was performed using Imaris 7.7.1 software (http://www.bitplane.com) as previous described^16,53^. Briefly, the *.oib* file was dragged and dropped into the main window of Imaris, and the *blend mode* was used to display the data. Then *Edit* menu was accessed and *Show Display Adjustment* was selected. Subsequently, the arrows were moved as needed to adjust each channel’s brightness and contrast of the images to optimize the visualization. Care was taken so as not to remove any data from the images. Images were processed with Adobe Photoshop CS6 (Adobe Systems, San Jose, CA, USA) and marked with Adobe Illustration CC 2019 (Adobe Systems, San Jose, CA, USA).

### Statistical analysis

The areas of the ischemic hemisphere and contralateral hemisphere were quantitatively analyzed using the analysis system of the panoramic tissue slice scanner. Three lower power images per rat (10 ×, 1260 μm × 1260 μm) from every 12th coronal brain sections were used to calculate the mean intensity of gP-CD31, mM-CD31, phalloidin and α-SMA labeling by using Image J (National Institutes of Health developed imaging processing program, Bethesda, Maryland, USA), respectively. Besides, the percentage of gP-CD31, Nissl, phalloidin and α-SMA labeling was also analyzed by Image J respectively. All data were expressed as mean ± standard error (SEM) and processed with GraphPad Prism version 8.2.1 (La Jolla, CA, USA). Two-tailed *t*-test was applied for the comparison between two groups, and a one-way analysis of variance (ANOVA) was carried out followed by Tukey test for multiple group comparisons. *P* < 0.05 was considered statistically significant.

### Ethical approval

This study was approved by the ethics committee of Institute of Acupuncture and Moxibustion, China Academy of Chinese Medical Sciences, Beijing, China (No. D2018-09-29-1). All experimental procedures were approved by the institutional and local committee on the care and use of animals. The study is reported in accordance with ARRIVE guidelines.

## Supplementary Information

Supplementary Information.

## Data Availability

The datasets used and/or analyzed during the current study available from the corresponding author on reasonable request.

## Abbreviations

α-SMA: Alpha smooth muscle actin
CD31: Cluster of differentiation 31
DAPI: 4′,6-Diamidino-2-phenylindole
gP-CD31: Goat polyclonal anti-CD31
mM-CD31: Mouse monoclonal anti-CD31
MCAO: Middle cerebral artery occlusion
PFA: Paraformaldehyde
PB: Phosphate buffer
PECAM-1: Platelet-endothelial cell adhesion molecule-1
TTC: 2,3,5-Triphenyltetrazolium chloride

## References

[1] Yao Y, Shaligram SS, Su H (2021). Brain vascular biology. Handb. Clin. Neurol..

[2] Liebner S, Plate KH (2010). Differentiation of the brain vasculature: The answer came blowing by the Wnt. J. Angiogenes Res..

[3] Wittko-Schneider IM, Schneider FT, Plate KH (2014). Cerebral angiogenesis during development: Who is conducting the orchestra?. Methods Mol Biol..

[4] Bär T (1983). Patterns of vascularization in the developing cerebral cortex. Ciba Found. Symp..

[5] Yang Y (2018). Vascular tight junction disruption and angiogenesis in spontaneously hypertensive rat with neuroinflammatory white matter injury. Neurobiol. Dis..

[6] Adams HP (2019). Cancer and cerebrovascular disease. Curr. Neurol. Neurosci. Rep..

[7] Kisler K, Nelson AR, Montagne A, Zlokovic BV (2017). Cerebral blood flow regulation and neurovascular dysfunction in Alzheimer disease. Nat. Rev. Neurosci..

[8] Privratsky JR, Newman PJ (2014). PECAM-1: Regulator of endothelial junctional integrity. Cell Tissue Res..

[9] Lertkiatmongkol P, Liao D, Mei H, Hu Y, Newman PJ (2016). Endothelial functions of platelet/endothelial cell adhesion molecule-1 (CD31). Curr. Opin. Hematol..

[10] Chen MB (2020). Brain endothelial cells are exquisite sensors of age-related circulatory cues. Cell Rep..

[11] Wang X (2021). Type 2 immunity induced by bladder extracellular matrix enhances corneal wound healing. Sci. Adv..

[12] Zhu D (2020). Macrophage M2 polarization induced by exosomes from adipose-derived stem cells contributes to the exosomal proangiogenic effect on mouse ischemic hindlimb. Stem Cell Res. Ther..

[13] Shen Y (2022). Histochemistry of microinfarcts in the mouse brain after injection of fluorescent microspheres into the common carotid artery. Neural Regen. Res..

[14] Shen Y (2022). Temporal alteration of microglia to microinfarcts in rat brain induced by the vascular occlusion with fluorescent microspheres. Front. Cell. Neurosci..

[15] Zhang S (2022). Temporal alterations in pericytes at the acute phase of ischemia/reperfusion in the mouse brain. Neural Regen. Res..

[16] Wang J (2020). A new approach for examining the neurovascular structure with phalloidin and calcitonin gene-related peptide in the rat cranial dura mater. J. Mol. Histol..

[17] Liu Y, Hu XB, Zhang LZ, Wang Z, Fu R (2021). Knockdown of arginyl-tRNA synthetase attenuates ischemia-induced cerebral cortex injury in rats after middle cerebral artery occlusion. Transl. Stroke Res..

[18] Lopez MS, Vemuganti R (2018). Modeling transient focal ischemic stroke in rodents by intraluminal filament method of middle cerebral artery occlusion. Methods Mol. Biol..

[19] Lee JY (2020). Eyeballing stroke: Blood flow alterations in the eye and visual impairments following transient middle cerebral artery occlusion in adult rats. Cell Transplant..

[20] Su X (2019). 'Governor vessel-unblocking and mind-regulating' acupuncture therapy ameliorates cognitive dysfunction in a rat model of middle cerebral artery occlusion. Int. J. Mol. Med..

[21] Alarcon-Martinez L (2018). Capillary pericytes express α-smooth muscle actin, which requires prevention of filamentous-actin depolymerization for detection. Elife.

[22] Wulf E, Deboben A, Bautz FA, Faulstich H, Wieland T (1979). Fluorescent phallotoxin, a tool for the visualization of cellular actin. Proc. Natl. Acad. Sci. U.S.A..

[23] Cooper JA (1987). Effects of cytochalasin and phalloidin on actin. J. Cell Biol..

[24] Wang BL, Larsson LI (1985). Simultaneous demonstration of multiple antigens by indirect immunofluorescence or immunogold staining. Novel light and electron microscopical double and triple staining method employing primary antibodies from the same species. Histochemistry.

[25] Robertson D, Savage K, Reis-Filho JS, Isacke CM (2008). Multiple immunofluorescence labelling of formalin-fixed paraffin-embedded (FFPE) tissue. BMC Cell Biol..

[26] Robertson D, Isacke CM (2011). Multiple immunofluorescence labeling of formalin-fixed paraffin-embedded tissue. Methods Mol. Biol..

[27] Asadi A, Bruin JE, Kieffer TJ (2015). Characterization of antibodies to products of proinsulin processing using immunofluorescence staining of pancreas in multiple species. J. Histochem. Cytochem..

[28] Yeon H, Cho Y, Seo J, Sim Y, Chang JB (2022). Simultaneous amplification of multiple immunofluorescence signals via cyclic staining of target molecules using mutually cross-adsorbed antibodies. Sci. Rep..

[29] Cui JJ (2022). Alexa Fluor 488-conjugated cholera toxin subunit B optimally labels neurons 3–7 days after injection into the rat gastrocnemius muscle. Neural Regen. Res..

[30] Wang J (2022). Sensory and autonomic innervation of the local tissues at traditional acupuncture point locations GB14, ST2 and ST6. Acupunct. Med..

[31] Lipman NS, Jackson LR, Trudel LJ, Weis-Garcia F (2005). Monoclonal versus polyclonal antibodies: Distinguishing characteristics, applications, and information resources. ILAR J..

[32] Kapingidza AB, Kowal K, Chruszcz M (2020). Antigen–antibody complexes. Subcell. Biochem..

[33] Han X (2020). Hapten-branched polyethylenimine as a new antigen affinity ligand to purify antibodies with high efficiency and specificity. ACS Appl. Mater. Interfaces.

[34] Fischman S, Ofran Y (2018). Computational design of antibodies. Curr. Opin. Struct. Biol..

[35] Perchiacca JM, Tessier PM (2012). Engineering aggregation-resistant antibodies. Annu. Rev. Chem. Biomol. Eng..

[36] Yang G, Velgos SN, Boddapati SP, Sierks MR (2014). Probing antibody–antigen interactions. Microbiol. Spectr..

[37] Vimer S, Ben-Nissan G, Marty M, Fleishman SJ, Sharon M (2021). Direct-MS analysis of antibody–antigen complexes. Proteomics.

[38] Blottière HM (1995). Utilization of activated U937 monocytic cells as a model to evaluate biocompatibility and biodegradation of synthetic calcium phosphate. Biomaterials.

[39] Nelson PN (2000). Monoclonal antibodies. Mol. Pathol..

[40] Vockova P (2021). CD31/PECAM-1 impacts engraftment, growth and spread of mantle cell lymphoma cells and positively correlates with extramedullary involvement. Leuk. Lymphoma.

[41] Li Y, Hoffman MD, Benoit DSW (2021). Matrix metalloproteinase (MMP)-degradable tissue engineered periosteum coordinates allograft healing via early stage recruitment and support of host neurovasculature. Biomaterials.

[42] Ilan N, Madri JA (2003). PECAM-1: Old friend, new partners. Curr. Opin. Cell Biol..

[43] Xie Y, Muller WA (1993). Molecular cloning and adhesive properties of murine platelet/endothelial cell adhesion molecule 1. Proc. Natl. Acad. Sci. U.S.A..

[44] Wan B (2021). GIT1 protects traumatically injured spinal cord by prompting microvascular endothelial cells to clear myelin debris. Aging.

[45] Quan X (2021). The role of LR-TIMAP/PP1c complex in the occurrence and development of no-reflow. EBioMedicine.

[46] Iadecola C (2017). The neurovascular unit coming of age: A journey through neurovascular coupling in health and disease. Neuron.

[47] Andreone BJ, Lacoste B, Gu C (2015). Neuronal and vascular interactions. Annu. Rev. Neurosci..

[48] Freitas-Andrade M, Raman-Nair J, Lacoste B (2020). Structural and functional remodeling of the brain vasculature following stroke. Front. Physiol..

[49] Profaci CP, Munji RN, Pulido RS, Daneman R (2020). The blood–brain barrier in health and disease: Important unanswered questions. J. Exp. Med..

[50] Liu M (2020). Cottonseed oil alleviates ischemic stroke injury by inhibiting the inflammatory activation of microglia and astrocyte. J. Neuroinflammation.

[51] Bian X, Ma K, Zhang C, Fu X (2019). Therapeutic angiogenesis using stem cell-derived extracellular vesicles: An emerging approach for treatment of ischemic diseases. Stem Cell Res. Ther..

[52] Navaratna D, Guo S, Arai K, Lo EH (2009). Mechanisms and targets for angiogenic therapy after stroke. Cell Adh. Migr..

[53] Wright GD, Horn HF (2016). Three-dimensional image analysis of the mouse cochlea. Differ. Res. Biol. Divers..

