# Author Correction: The immunolocalization of cluster of differentiation 31, phalloidin and alpha smooth muscle actin on vascular network of normal and ischemic rat brain

**DOI:** 10.1038/s41598-023-29086-x

**Published:** 2023-01-31

**Authors:** Jia Wang, Yating Guo, Dongsheng Xu, Jingjing Cui, Yuqing Wang, Yuxin Su, Yihan Liu, Yi Shen, Xianghong Jing, Wanzhu Bai

**Affiliations:** Institute of Acupuncture and Moxibustion, China Academy of Chinese Medical Sciences, Beijing, 100700 China

Correction to: *Scientific Reports* 10.1038/s41598-022-26831-6, published online 24 December 2022

The original version of this Article contained an error in the Funding section.

“This research was funded by the National Natural Science Foundation of China (No. 82004299); and the CACMS Innovation Fund (No. CI2021A03407); and the Fundamental Research Funds for the Central Public Welfare Research Institutes (No. ZZ14-YQ-032; ZZ14-YQ-034; ZZ13-YQ-068).”

now reads:

“This research was funded by the National Natural Science Foundation of China (No. 82004492); and the CACMS Innovation Fund (No. CI2021A03407); and the Fundamental Research Funds for the Central Public Welfare Research Institutes (No. ZZ14-YQ-032; ZZ14-YQ-034; ZZ13-YQ-068).”

The original Article has been corrected.

