# Supplementary material for: The immunolocalization of cluster of differentiation 31, phalloidin and alpha smooth muscle actin on vascular network of normal and ischemic rat brain
Source: Sci Rep. 2022 Dec 24;12:22288. doi: 10.1038/s41598-022-26831-6 (PMC9789995; doi:10.1038/s41598-022-26831-6)
Supplement: Supplementary file 1 — Supplementary Information. [file 41598_2022_26831_MOESM1_ESM.pdf]

Research article

The immunolocalization of cluster of differentiation 31, phalloidin and alpha smooth muscle actin on vascular network of normal and ischemic rat brain

Jia Wang[1,†], Yating Guo[1,†], Dongsheng Xu[1], Jingjing Cui[1], Yuqing Wang[1], Yuxin Su[1], Yihan Liu[1], Yi Shen[1], Xianghong Jing[1], and Wanzhu Bai[1,*]

1 Institute of Acupuncture and Moxibustion, China Academy of Chinese Medical Sciences, Beijing, China, 100700
* Correspondence: Wanzhu Bai, Institute of Acupuncture and Moxibustion, China Academy of Chinese Medical Sciences; Email: wanzhubaisy@hotmail.com.
† These authors contributed equally to this work.

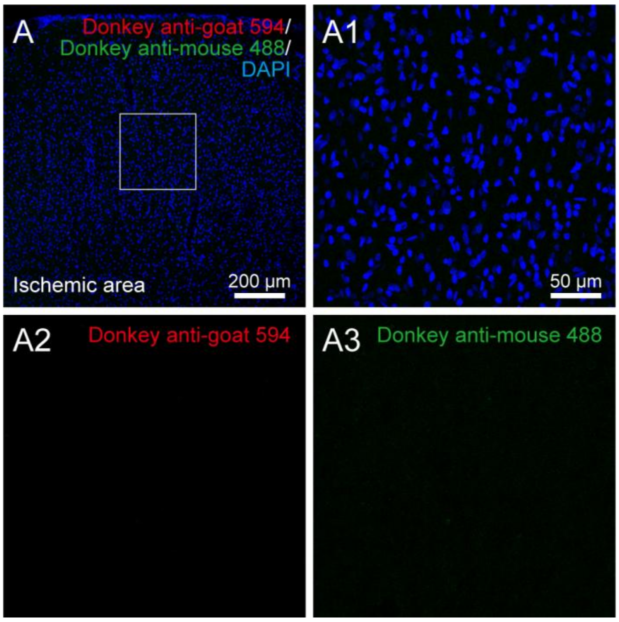

Supplementary Figure 1. Distribution of secondary antibodies donkey anti-goat Alexa Fluor (AF) 594 and donkey anti-mouse AF488 in the rat brain with ischemia/reperfusion.

A: Representative photograph from the cerebral cortex directly labeled with donkey anti-goat AF594 and donkey anti-mouse AF488 showing there was no positive expression. A1-A3: Corresponding magnified photograph from panel A (box-indicated region) showing the detailed labeling (A1) separately with donkey anti-goat AF594 (A2) and donkey anti-mouse AF488 (A3). The cellular nucleus was stained by DAPI (blue). Scale bars: 200 μm in A, 50 μm in A1-A3.

Supplementary Table 1. Application of gP-CD31 and mM-CD31 in previous studies

References	Antibody	Animal	Model	Finding	Vascular network
Shen 2022 [1]	gP-CD31	Rat	Multiple cerebral infarctions	Under normal circumstances there was a modest expression of CD31-labeled capillaries. Following surgery, the necrosis of blocked arteries and the proliferation of surrounding capillaries were carried out over time. CD31-labeled capillaries were abundantly expressed surrounding the infarcted vessels.	Yes
Zhang 2022 [2]	gP-CD31	Mouse	Ischemia/reperfusion	The sham operation group had unconstricted microvessels near the PDGFR-β-positive pericytes and no pathological changes. After 2, 12, and 24 hours of cerebral ischemia/reperfusion, the morphology of the CD31-labeled capillaries near the pericyte cell bodies changed, with distortion and even narrowing of the tube diameter, which positively correlated with the reperfusion time.	Yes
Wang 2021 [3]	gP-CD31	Mouse	Corneal injury	CD31 revealed the vasculature in the limbus area after injury and treatment. In comparison with the relatively dim staining of CD31 in no injury controls, PBS-treated corneas demonstrated an increase of vasculature in the limbus area at 1 and 2 weeks after surgery, consistent with chronic inflammation and possible neovascularization. Micro-UBM–treated corneas showed some increase of vasculature in the limbus area at 1 week after surgery, but by week 2, the extent of vascularization decreased. The vasculature in the UF-UBM group remained low at both 1 and 2 weeks after injury.	Yes
Chen 2020 [4]	gP-CD31	Mouse	Aged model	CD31-labeled vascular networks in hippocampus of young (3 months) and aged (20 months) mice were provided with no quantification.	Yes
Zhu 2020 [5]	gP-CD31	Mouse	Hindlimb ischemia	Increases were observed for the vascular endothelial cell marker CD31 in the adductor muscle and the gastrocnemius muscle	Yes
Shen 2022 [6]	mM-CD31	Mouse	Multiple cerebral infarctions	CD31 labeling expressed more strongly in the regions of microinfarct than that of control.	No
Wang 2020 [7]	mM-CD31	Rat	Normal rats	CD31-labeled vessels were located on the distal part of blood vessels similar to capillaries in morphology, while CD31-labeled vessels showed only the vessel outline, not the whole lumen.	No
Xu 2019 [8]	mM-CD31	Rat	Stress	Compared with the control rats, the results indicated decreased ratio of GLUT-1 fluorescence intensity/CD31 fluorescence intensity in the restraint-stressed rats at 7, 14 and 21 days.	No
Gandin 2016 [9]	mM-CD31	Mouse	Ischemia/reperfusion	Treatment with MLC901 significantly increased the CD31-positive capillary density, vascular perimeters and BrdU-reactive endothelial cell numbers in the ischemic border, as compared with the vehicle-treated mice. Double immunostaining BrdU/CD31 showed that BrdU-positive endothelial cells localized in the vessel, indicating that MLC901 enhanced endothelial cell proliferation and angiogenesis by increasing the number of neocortical vessels invading the ipsilateral infarcted area.	No
Eckert 2015 [10]	mM-CD31	Mouse	Ischemia/reperfusion	Only showed CD31-labeled blood vessels but without a detailed description and statistical analysis.	No

Kleinschnitz 2011 [11]	mM-CD31	Mouse	Ischemia/reperfusion	Double labeling for glucocorticoid receptor (GR) and CD31 could identify cerebral blood vessels as a major source of GR protein in the murine brain. However, there was no detailed description and statistical analysis of CD31-labeled vessels.	No

References

1 Shen, Y. *et al.* Temporal alteration of microglia to microinfarcts in rat brain induced by the vascular occlusion with fluorescent microspheres. *Frontiers in cellular neuroscience* **16**, 956342, doi:10.3389/fncel.2022.956342 (2022).

2 Zhang, S. *et al.* Temporal alterations in pericytes at the acute phase of ischemia/reperfusion in the mouse brain. *Neural regeneration research* **17**, 2247-2252, doi:10.4103/1673-5374.336876 (2022).

3 Wang, X. *et al.* Type 2 immunity induced by bladder extracellular matrix enhances corneal wound healing. *Science advances* **7**, doi:10.1126/sciadv.abe2635 (2021).

4 Chen, M. B. *et al.* Brain Endothelial Cells Are Exquisite Sensors of Age-Related Circulatory Cues. *Cell reports* **30**, 4418-4432.e4414, doi:10.1016/j.celrep.2020.03.012 (2020).

5 Zhu, D. *et al.* Macrophage M2 polarization induced by exosomes from adipose-derived stem cells contributes to the exosomal proangiogenic effect on mouse ischemic hindlimb. *Stem cell research & therapy* **11**, 162, doi:10.1186/s13287-020-01669-9 (2020).

6 Shen, Y. *et al.* Histochemistry of microinfarcts in the mouse brain after injection of fluorescent microspheres into the common carotid artery. *Neural regeneration research* **17**, 832-837, doi:10.4103/1673-5374.322470 (2022).

7 Wang, J. *et al.* A new approach for examining the neurovascular structure with phalloidin and calcitonin gene-related peptide in the rat cranial dura mater. *Journal of molecular histology* **51**, 541-548, doi:10.1007/s10735-020-09903-7 (2020).

8 Xu, G. *et al.* Restraint Stress Induced Hyperpermeability and Damage of the Blood-Brain Barrier in the Amygdala of Adult Rats. *Frontiers in molecular neuroscience* **12**, 32, doi:10.3389/fnmol.2019.00032 (2019).

9 Gandin, C., Widmann, C., Lazdunski, M. & Heurteaux, C. MLC901 Favors Angiogenesis and Associated Recovery after Ischemic Stroke in Mice. *Cerebrovascular diseases (Basel, Switzerland)* **42**, 139-154, doi:10.1159/000444810 (2016).

10 Eckert, A. *et al.* Bystander Effect Fuels Human Induced Pluripotent Stem Cell-Derived Neural Stem Cells to Quickly Attenuate Early Stage Neurological Deficits After Stroke. *Stem cells translational medicine* **4**, 841-851, doi:10.5966/sctm.2014-0184 (2015).

11 Kleinschnitz, C. *et al.* Glucocorticoid insensitivity at the hypoxic blood-brain barrier can be reversed by inhibition of the proteasome. *Stroke* **42**, 1081-1089, doi:10.1161/strokeaha.110.592238 (2011).
